# RETRACTION: Alternative Pathways of Cancer Cell Death by Rottlerin: Apoptosis versus Autophagy

**DOI:** 10.1155/ecam/9859127

**Published:** 2026-06-29

**Authors:** Evidence-Based Complementary and Alternative Medicine

RETRACTION: C. Torricelli, S. Salvadori, G. Valacchi, et al., “Alternative Pathways of Cancer Cell Death by Rottlerin: Apoptosis versus Autophagy,” *Evidence-Based Complementary and Alternative Medicine*, 2012, 980658, https://doi.org/10.1155/2012/980658.

The above article, published online on 20 December 2012 in Wiley Online Library (https://wileyonlinelibrary.com), has been retracted by John Wiley & Sons Ltd. The retraction has been agreed due to multiple concerns identified in the western blots presented in Figure 2. Specifically:

• In Figure 2a, the first and fourth PARP band appear highly similar.
• In Figure 2a, the first and sixth Procaspase 9 band appear identical.
• In Figure 2a, the second and sixth Procaspase 9 band appear highly similar when flipped horizontally.
• In Figure 2a, lanes 1–3 of the bottom‐most ß‐actin bands appear highly similar to lanes 3–5 of the ß‐actin bands as shown in Figure 4c.
• In Figure 2a, the PARP and Cleaved‐PARP bands show evidence of undeclared spicing.
• In Figure 2a, after resizing, the lanes 2–5 of the first set of ß‐actin bands appear highly similar to the ß‐actin bands as shown in Figure 2 of [1].

The authors provided a response to the concerns but could not provide the original western blot images. After assessment of the concerns, the results and conclusions can no longer be considered reliable, and the article is therefore retracted.

Karel Soucek disagrees with the retraction, whilst the remaining authors remained unresponsive.
